# Merkel Cell Carcinoma: Epidemiology, Target, and Therapy

**DOI:** 10.1007/s13671-014-0068-z

**Published:** 2014-01-22

**Authors:** Mathew P. Hughes, Matthew E. Hardee, Lynn A. Cornelius, Laura F. Hutchins, Jurgen C. Becker, Ling Gao

**Affiliations:** 1 Department of Dermatology, University of Arkansas for Medical Sciences (UAMS), 4301 W. Markham St., # 576, Little Rock, 72205 USA; 2 Department of Radiation Oncology, UAMS, 4301 W. Markham St., # 771, Little Rock, 72205 USA; 3 Department of Internal Medicine, Division of Dermatology, Washington University School of Medicine in St. Louis, St. Louis, 63110 USA; 4 Department of Medicine, UAMS, 4301 W. Markham St., # 508, Little Rock, 72205 USA; 5 General Dermatology and Immunology, Medical University of Gaze, Auenbruggerplatz 8, 8036 Graz, Austria

**Keywords:** Merkel cell carcinoma, Merkel cell polyomavirus, Immunosuppression, Sentinel lymph node biopsy, Immune therapy, Chemotherapy, Radiation therapy, Signaling pathway

## Abstract

Merkel cell carcinoma (MCC) is an aggressive neuroendocrine tumor of the skin with a rising incidence. MCC has metastatic potential regardless the size of the primary tumor and a 5-year disease associated mortality rate is 46 %. Surgery and radiation are the mainstays of management for primary MCC. There is no evidence-based effective chemotherapy for recurrent or metastatic diseases to date. In-depth mechanistic studies in MCC have uncovered important cellular events and the association with a polyomavirus, which has provided direct evidence for molecular targeted and immunotherapy. Further perspective studies and clinical trials are warranted to provide reliable evidence of possible pitfalls and effectiveness of molecular targeted immunotherapy alone or in combination with chemotherapy in MCC.

## Introduction

Merkel cell carcinoma (MCC) is a rare neuroendocrine tumor of the skin with a rising incidence and an aggressive behavior. It was first described as trabecular carcinoma in 1972 by Toker [1]. The name of MCC was adopted because the neoplasm was originally thought to arise from the Merkel cells of the epidermis [2]. However, recent observations question the notion and propose that pluripotent dermal stem cells are the origin of MCC [3]. Interestingly, early B cell differentially is also identified in MCCs [4]. Currently, surgical excision and radiotherapy are the mainstays of management for primary MCCs. Although one-third of the patients will eventually develop distant metastasis, there is no evidence-based effective chemotherapy for recurrent and metastatic disease with a proven survival benefit. Recently, a causal link between the Merkel cell polyomavirus (MCV) and the pathogenesis of MCC, together with both MCV dependent and independent cellular events, holds the promise for mechanism-based and disease specific therapy. This is a review of the epidemiology, immune targets and therapy of MCC with a focus on recent literature.

## Epidemiology

### Incidence and Mortality

The annual incidence of MCC is 0.6 per 100,000 persons and is increasing (approximately 1,600 new cases per year in the US) [5•]. In Australia, between 1986–2001, the age-adjusted incidence rose at an annual increase of 8 %, as compared to the 3 % rise in cutaneous melanoma [6]. In the Netherlands, the incidence per million increased from 1.7 in 1993–1997 to 3.5 in 2003–2007 [7]. This rising incidence is partly due to increased awareness and improved diagnostic techniques, especially the introduction of cytokeratin 20 immunostaining. The median age at diagnosis is 76.2 years for women and 73.6 years for men. Only 4 % of patients are diagnosed at 49 years or younger, and it is exceedingly rare in children with only scattered case reports [8•]. The incidence of MCC is approximately 11-fold to 13-fold greater in patients with AIDS and 5-fold to 10-fold greater for people with a solid organ transplant [9]. According to the National Cancer Data Base (NCDB), the majority of MCCs present with local disease (66 %), followed by nodal disease (27 %) and metastatic disease (7 %). However, the percentage of patients with nodal disease is higher if a sentinel lymph node biopsy (SLNB) is performed. Patients with local disease had a 64 % relative survival at five years, as compared to 39 % in regional nodal disease and 18 % in metastatic disease [10••].

### UV Exposure and Immunosuppression

There is a positive association between geographic UVB radiation indices and age-adjusted MCC incidence among white patients [6]. Moreover, the incidence of MCC was 100 times greater in patients with a history of PUVA treatment than that in the general population [11]. According to data collected from the Surveillance, Epidemiology and End Results (SEER) from 1973 to 2006, 94.9 % of patients were white with tumors in the head and neck region. African Americans represented only 1 % of patients, with tumors arising more frequently on the lower extremities [8•]. Similar to melanoma, another UV-induced tumor, MCC may have a predilection for the left face and arms [12]. Nonetheless, MCCs also occur on non-sun-exposed areas such as on the buttocks. Immunosuppression is a known risk factor for the development of MCC. More specifically, immunosuppressed patients with T-cell dysfunction, i.e., solid organ transplant recipients, AIDS patients and chronic lymphocytic leukemia patients are at an increased risk of developing MCC. Further observations highlighting the importance of the immune system are cases of MCC regressing following improvement in immune function and sudden clearance of MCC after recognition by the immune system [13, 14].

### Merkel Cell Polyomavirus (MCV)

Polyomaviruses are small, nonenveloped, double-stranded, circular DNA viruses. The initial association of MCV and MCC was described by Chang and colleagues in 2008 using Digital Transcriptome Subtraction and cDNA libraries created from MCC tumor mRNA [15]. Since then, several groups have independently verified an association between MCV and MCC.

#### MCV Prevalence

In the general population, MCV seroprevalence is 9 % in children younger than four years of age and increases to 35 % by 4–13 years of age [16•]. Using immunoassays, Tolstov et al., found that 80 % of healthy North American adults (blood donors) showed evidence for past MCV exposure. Consistent with this, MCV was detected in 80 % of cutaneous swabs from healthy volunteers, suggesting it may be a common inhabitant of the human skin [17]. Despite this prevalence, extensive efforts sought to find an association between MCV and other diseases have largely failed [16•].

#### Pathogenesis

Although MCV is strongly associated with MCC and many studies support its role in pathogenesis, the presence of virus is not sufficient to induce MCC carcinogenesis. As with other cancer-associated viruses, additional cellular events together with loss of immunosurveillance are postulated to contribute to MCC tumor development. Thus, although infection with MCV is common, MCC is still a very rare cancer. MCV encodes a large T tumor antigen (LT) and a small antigen tumor (sT), which both play a role in MCC pathogenesis by targeting several tumor suppressor genes. (18). Since the discovery of MCV, both LT and sT have been used as biomarkers, aid in predicting prognosis, to gauge therapeutic response and in the detection of recurrence of MCC. However, the pathogenic role of MCV needs further elucidation. For example, the dependence of MCC cell lines on LT and/or sT differs. Similarly, studies have yielded conflicting results in terms of the presence of MCV and viral load on clinical implications in MCC [18].

## Therapeutic Targets

### Host Immune System

Epidemiologic data suggest a strong link between MCC and the immune system. MCV infection induces a humoral and cellular immune response, notably, while immune responses to the viral capsid proteins are present in most people, immune responses to the transforming early genes, i.e., LT and sT, are only present in MCC patients. A transcriptome study of MCC tumors demonstrates a positive association between improved outcomes and robust infiltration of CD8+ lymphocytes in the tumor [19]. Moreover, a recent study has found the MCV-specific T cells express high levels of immune checkpoint receptor PD1 and Tim-3, which provide direct evidence of immune therapy in MCCs [5•].

### Aberrant Signaling Pathways in Tumor Cells

Cytotoxic activity by chemotherapy has been the mainstay in the management of advanced MCC. However, it failed to demonstrate improved survival and is associated with a high mortality rate [20, 21]. Until recently, aberrations in signal transduction contributing to the oncogenic phenotype of MCC were largely unknown. However, in-depth mechanistic studies are necessary to provide the rationale for mechanism-based antitumor therapy. Interrogation of MCC tumors for mutations of both tumor suppressor genes and oncogenes, such as p53, PTEN, Ras, B-RAF, c-kit, β-catenin, which are frequently mutated and dysregulated in many cancers, have failed to reveal a consistent significant role for any of the genes in MCC [22]. More specifically, in search of receptor tyrosine kinase (RTK) involvement in MCC tumorigenesis (providing a rationale for the use of targeted therapies), studies have found variable expression of c-kit, VEGFs, PDGFα and PDGFβ in MCCs compared to normal skin. Intriguingly, one study has shown that the MAP kinase pathway is silent (as demonstrated by lack of pathway activation and no ERK phosphorylation) in the majority of MCCs examined [23]. Recently, the PI3K/AKT and mTOR pathway, the most common dysregulated pathway in human cancer, was found to be up-regulated in MCCs, despite low mutation rates of PI3K/Akt detected [24•, 25, 26]. Collectively, activated pathways will be potential targets in mechanism-based therapy.

## Staging and Treatment

The American Joint Committee on Cancer TNM Staging Classification should be used for a comprehensive staging system of MCC patients. MCC localized to the skin are staged according to the size of the primary tumor- stage I (≤ 2 cm) or stage II (>2 cm). The A and B subclassification is based on pathological versus clinical evaluation of lymph nodes. Stage III is a regional nodal disease with stage IIIA being positive lymph nodes identified by pathology and stage IIIB being clinically or radiological positive lymph nodes. Stage IV is metastatic disease outside of the regional nodal basin.

### Surgery

#### Wide Local Excision vs. Mohs Micrographic Surgery

Based on the current National Comprehensive Cancer Network (NCCN) guidelines, wide local excision is the mainstay treatment for stage I and stage II diseases. Wide local excision with a 2 to 3 cm margin is recommended, except for the head and neck region where narrow margins may be acceptable. There is limited data regarding the local recurrence rate between wide local excision and Mohs surgery. It is important to note that the clinical presentations for locoregional recurrence are satellite and in-transit metastases. Therefore, Mohs surgery is challenged in MCC as per continuitatem invasion is the prominent cause for recurrences. Thus, the central portion of the tumor is recommended for permanent sections if Mohs micrographic surgery is used.

### Sentinel Lymph Node biopsy (SLNB

SLNB is recommended in both Stage I and Stage II diseases, due to the metastatic potential of MCC (10). A study by Bichakjian, et al., found a SLNB positivity rate of 20 %-30 % in patients with Stage I disease and clinically negative node [27]. Furthermore, SLNB-negative patients carry a better prognosis than patients with only clinical nodal evaluation. In a study including 5,823 MCC patients, those with localized-disease, as well as pathologically negative nodes on SLNB had a significantly better outcome than those undergoing only clinical nodal assessment [10••]. SLNB results in more accurate staging, and if positive, nodal treatment decreases recurrence. However, SLNB is not recommended if it will not change management or if the prognostic data does not result in a change of management of the patient. Although studies comparing radiation alone with complete lymph node dissection yields similar outcomes, the most common practice for MCC patients with a positive SNL is complete lymph node dissection in conjunction with radiation.

### Radiation

#### Fractionated

Standard postoperative treatment consists of 50–60 Gy given in conventional equal fractions over a 5–6 week period [28]. Although adjuvant radiation therapy is controversial for stage I and stage II MCC, it is recommended by NCCN for advanced local and regional disease. All published studies on the efficacy of adjuvant radiation have been retrospective. Most of these studies have demonstrated an increase in locoregional control and improvement in disease-free survival with postoperative radiation therapy [29], but not in all cases [30, 31]. A literature review by Medina-Franco et al., demonstrated a significantly decreased rate of local recurrence (10.5 %) with adjuvant radiotherapy compared to surgical excision alone (52.6 %) [32]. Two recent studies have also demonstrated improved overall survival with postoperative radiation therapy [27, 33]. Elective radiation therapy to regional nodal basins has been used to decrease the regional recurrence rate. A randomized trial of elective nodal irradiation versus observation in early stage MCC was terminated early; however, analysis of accrued patients revealed a significant decrease in regional recurrence in the radiotherapy arm (0 % vs. 16.7 %) [34].

Radiotherapy may be used alone for MCC when the tumor is inoperable or when the patient refuses surgery. Interestingly, no statistical difference was found in overall and disease-free survival in 25 patients with primary MCC treated with radiotherapy alone as compared to patients treated with surgery followed by radiotherapy [32]. In evaluation of radiation as a monotherapy for lymph node-positive MCC, Fang et al., found that there was no difference with regard to overall survival between radiotherapy and completion lymphadenectomy [35]. A similar control rate has been reported by Sundaresan et al., a definitive radiation treatment of 30 regions (primary and regional nodes) resulted in a two-year local control rate of 89 % [36].

#### Surface-mold Computer-Optimized Brachytherapy

In-transit metastases (Stage IIIB disease) usually develop in the extremities and clinically appear to be violaceous nodules or palpable subcutaneous nodules. Because in-transit metastases are typically multifocal, conventional external-beam radiation therapy can be technically challenging. Surface-mold computer-optimized brachytherapy (SMBT) allows delivery of high doses of radiation achieving an effective local control with limited toxicity. A recent report by Wang et al., of ten MCC patients and a total of 152 in-transit metastases were treated with high dose radiation (12 Gy in two equal fractions of radiation). Although high dose SMBT brachytherapy did not alter the disease course with respect to overall survival, it provided a good in-field control rate of 99 % [35].

#### Emerging Radiation Techniques

A single fraction of high-dose radiation has been showed to stimulate lymph node priming and reduce primary and metastatic tumors in a CD8 T-cell – mediated fashion in a melanoma mouse model [37]. Treatment with high doses and fewer fractions has been safe and effective in palliation, as well as more convenient for patients. In a study by Nghiem’s group including 15 MCC patients treating with a single fraction of 8 Gy to lymph node or internal organ metastases, they reported 11 complete and four partial responses in a median follow-up of five months [38]. No significant side effects were observed. This palliative regimen is particularly attractive to elderly patients who depend on transportation, with the possibility of combining treatment of symptomatic lesion with a potential stimulation of immune response to MCC. However, long-term follow-up data is needed before it becomes a mainstream approach.

In sum, radiation is beneficial as an adjuvant therapy for advanced local and regional disease. However, questions regarding the role of radiation in early stage disease with negative SLNB and the potential for shortening radiation courses with higher dose per fraction remain.

### Chemotherapy

#### Systemic Chemotherapy

Although MCC is considered to be chemotherapy sensitive with initial efficacy in disease regression, resistance usually develops after the first 2–3 cycles, and it is associated with significant toxicity. In a retrospective study, 69 % of patients with locally advanced disease and 57 % with metastatic disease responded to first-line chemotherapy. However, survival was limited to an average of 24 months with locally advanced and nine months with metastatic disease; 7.7 % of deaths were associated with drug related toxicity [20, 21]. The most common cytotoxic agents used are platinum with or without etoposide. Others are anthracyclines, antimetabolites, cyclophosphamide, etoposide and platinum agents alone or in combination.

#### Isolated Limb Perfusion and Infusion

Isolated limb perfusion was developed in the 1950s to treat in-transit, unresectable melanoma of the extremities. The advantage is local delivery of chemotherapy to minimize systemic side effects. Subsequently, isolated limb infusion (ILI) was adopted in the early 1990s because it is safer and less invasive. Available data are largely based on extrapolation from the melanoma literature. Wong et al., recently reported their experience of over 100 ILI, including 79 melanoma patients and three MCC patients [39]. Actinomycin-D and melphalan were used and was dosed based on limb volume. At three months, the initial complete response rates for upper extremity and lower extremity melanoma are 42 % and 35 %, respectively. No significant side effects were reported. Although not commonly used for local and regional control of MCC, this treatment modality may prove beneficial in select MCC patients and further study is warranted.

### Immunotherapy

#### Cytokine Therapy

It is known that cytotoxic T-cell activation is required for antitumor activities. Cytokines are grouped as type I which is associated with cytotoxic T-cell activation, while type II is associated with antibody formation [40]. Although both type I and type II interferon induces apoptosis in MCV positive MCC cells in vitro, the clinical application of interferon α in two MCV positive MCC patients failed to demonstrate therapeutic efficacy [41, 42]. Of note, intralesional interferon β application shows early success in local control; however, further studies are needed. An ongoing phase II clinical trial is testing intralesional delivery of IL-12 plasmid followed by electroporation to promote Th1 response and increase interferon γ expression (http://clinicaltrials.gov/show/NCT01440816).

#### Adoptive Immunotherapy

This approach remains investigational, but it is associated with long-term remission. It involves harvesting tumor infiltrating lymphocytes or peripheral T cells, followed by ex vivo expansion. Further manipulation of tumor-reactive T-cells and co-administration with NK cells or dendritic cells and/or vaccine has been explored in melanoma. Hopefully MCC specific clinical trials are forthcoming soon. Additionally, the discovery of MCV renders a potential target for developing viral antigen directed immunotherapy or vaccine. A Phase I/II clinical trial is current recruiting metastatic MCC patients who will be treated with autologous T cells and IL-2 (aldesleukin) (http://clinicaltrials.gov/show/NCT01758458). Transgenic T-cell receptor based therapy and soluble T-cell antigen receptor (STAR) reagent has been also tested in other advance solid tumors.

#### Immune Checkpoint Inhibition

Comprehension of immune system modulation by targeting co-inhibitory and co-stimulatory receptors has become a promising new approach of immunotherapy for cancer. CTLA-4 is an important negative regulator of T-cell-mediated antitumor responses. Development of blocking antibodies directed against CTLA-4, such as ipilimumab, has opened a new era in the field of immunotherapy for cancer. Inspired by the success in melanoma, a randomized clinical trial is currently enrolling in Europe with ipilimumab versus observation following surgical resection of MCC (EUDRACT: 2013-000043-78). Similarly, another new molecule has been generated against programmed death-1 (PD1; CD279), an inhibitory receptor that down-regulates T cell function following engagement of PD1 ligand (PDL1) that is exclusively expressed on tumor cells. While CTLA-4 is only expressed in T cells, PD-1 expression is not only found in T cells, but also inducible in B-cell and natural killer (NK) cells. In order to block the inhibitory PD1 pathway, anti-PD1 and anti-PDL1 monoclonal antibodies have been generated with reportedly improved safety profiles and fewer side effects than as compared to anti-CTLA-4 antibodies. It is promising that another immune checkpoint modulating antibody 4-1BB (CD137) is currently in clinical development.

#### IMGN901 (BB10901, huN901-DM1)

CD56 is expressed on virtually all MCC tumors. IMGN901 is a novel CD56-targeting anti-cancer agent that consists of a potent cytotoxic agent, DM1, which is attached to a CD56-binding monoclonal antibody, huN901, using an engineered linker. Once bound to CD56, IMGN901 is internalized into the cancer cell and the DM1 is released, killing the cell via inhibition of the polymerization of tubulin. There is an ongoing Phase I clinical trial evaluating IMGN901 in CD56 expressing tumors, including MCC (http://clinicaltrials.gov/show/NCT00346385).

### Molecular Targeted Therapy

In parallel, efforts have showcased the efficacy of targeted therapy inhibiting disease-driving mutations of the BRAF and MAPK pathway inhibitors in melanoma.

#### PI3K Inhibitors

Up-regulation of PI3K/Akt has been demonstrated in two independent studies [25, 26], providing a rationale for inhibition of PI3K in MCC. There are several ongoing PI3K inhibitor clinical trials; however, none of them is MCC specific.

#### mTOR Inhibitors

The mammalian target of rapamycin (mTOR) pathway is a master regulator of protein synthesis and is frequently found to be dysregulated in human cancers. Likewise, mTOR is found to be up-regulated in MCC (18). The mTOR resides in two complexes, mTOR complex1 (mTORC1) and mTOR complex 2 (mTORC2), which execute distinct cellular tasks. Rapamycin and its analogues are allosteric inhibitors via mTORC1 inhibition. Sirolimus, temsirolimus, everolimus and deforolimus are also members of this family. Underscored by the clinical inefficacy of allosteric inhibitors, more potent inhibitors of the active site of mTOR kinase, such as PP242, WYE-354, Ku-0063794 and INK128, have been developed. INK128 (MLN0128) is currently open for Dose Escalation study in patients with advanced solid tumors based on its high potency (http://clinicaltrials.gov/show/NCT01058707).

#### Apoptosis Induction

Decreased apoptosis is evident in MCC regardless of MCV status. YM-155 has been shown to down regulate survivin expression and promote apoptosis in MCC xenograft tumors [43] Phase II clinical trials of YM-155 in combination of other agents have been completed for other tumor types. Other apoptotic inhibitors, such as ABT-263, have also shown some clinical efficacy as a single agent or in combination [44]. A Bcl-2 antisense has shown to halt tumor growth in MCC xenograft animal models, however, a Bcl-2 antisense, Genasense, fails to show therapeutic efficacy in MCC [45–47].

### Receptor Tyrosine Kinase Inhibitors

#### Imatinib

Although mutation in c-kit and PDGFR are rare events in MCCs, successful MCC patients treated with imatinib have been reported [48–50]. However, a Phase II clinical trial concluded that most MCCs progressed during the one or two cycles of treatment [51]. Therefore, there is limited clinical application of Imitinib in MCC.

#### Pazopanib

Pazopanib targets both VEGFR and PDGFR, which are overexpressed in a subset of MCC [49]. In one report, pazopanib resulted in a complete resolution of primary tumor of the scalp disease and a partial response in lung metastases [52]. Currently, a Phase II clinical trial for metastatic MCC is open in Europe (EUDRACT Number: 2011-003226-27).

### Octreotide

Octreotide is a potent analog of somatostatin and has anti-proliferative and anti-angiogenic effects in neuroendocrine tumors. Overexpression of somatostatin receptor 2 has been demonstrated at the mRNA level in 90 % MCC [53]. Moreover, a radiolabeled somatostatin analogue containing the active octapeptide of somatostatin (90Y-DOTATOC) induces remission in a metastatic MCC [54]. Furthermore, synchronous use of ^177^ lutetium-labeled somatostatin analogs and radiosensitizing chemotherapy in a MCC patient with visceral metastasis demonstrates the feasibility of applying this technique in metastatic MCCs. Based on this, a 177Lutetium-DOTA-Octreotate therapy in somatostatin receptor-expressing Neuroendocrine Neoplasms is recruiting patients (http://clinicaltrials.gov/show/NCT01237457).

## Conclusion

A hallmark of human cancer is heterogeneity. At the genetic level, cancer is a reflection of the complex series of changes resulting in the activation of oncogenes coupled with inactivation of tumor suppressor genes. However, targeted therapy at the gene level remains a challenge, as there is a distinction between driver mutations that can propel the development of cancer and driver mutations on which the cancer cell continually depends. Moreover, secondary resistance often develops, as observed in melanoma with BRAF inhibition. Given individual variation in the host immune system as manifested by disease outcome, response to therapy and ability to metastasize, combination therapy with immunomodulator becomes crucial. Recent advances in targeted therapy and immunotherapies in the treatment of metastatic melanoma have instigated application of a similar approach to other tumor types, including MCC. Therefore, researchers await Phase I and Phase II studies with great interest for MCC, as there is a great need for improved therapy.
